# Mechanical Properties of Poly(ethylene-co-methacrylic acid) Reinforced with Carbon Fibers

**DOI:** 10.3390/polym13010165

**Published:** 2021-01-05

**Authors:** Tatjana Haramina, Daniel Pugar, Darko Ivančević, Ivica Smojver

**Affiliations:** Faculty of Mechanical Engineering and Naval Architecture, University of Zagreb, I. Lučića 5, 10 000 Zagreb, Croatia; dpugar@fsb.hr (D.P.); divancevic@fsb.hr (D.I.); ismojver@fsb.hr (I.S.)

**Keywords:** E/MAA, composite, carbon fibers, mechanical properties, self-healing, dynamic mechanical analysis, ballistic tests, thermal history, compression molding

## Abstract

The capability of poly(ethylene-co-methacrylic acid) (E/MAA) to self-heal is well known, however, its mechanical properties are weak. In this study, composites with single and double layers of unidirectional (UD) carbon fibers were prepared by compression molding. Even a low mass fraction of fibers substantially improved the polymer. The flexural and tensile properties were tested at 0°, 45° and 90° fibers direction and compared to those of the matrix. The mechanical properties in the 0° direction proved superior. Flexural properties depended on the reinforcement distance from the stress neutral plane. The tensile modulus in the 0° direction was 13 times greater despite only a 2.5% mass fraction of fibers. However, both tensile modulus and strength were observed to degrade in the 90° direction. Dynamic mechanical analysis showed the dependence of both structure and properties on the thermal history of E/MAA. Tensile tests after ballistic impact showed that the modulus of the self-healed E/MAA was not affected, yet the strength, yield point, and particularly the elongation at break were reduced. A composite with higher fiber content could be prepared by mixing milled E/MAA particles in fibers prior to compression.

## 1. Introduction

Given their unique ability to self-heal ballistic damage, materials based on the copolymer of ethylene and methacrylic acid (E/MAA) are recognized as having enormous potential for a wide range of uses in containment applications, such as aircraft fuel tanks, protective barrier in oil tankers, pressure vessels, etc. [1,2]. Unfortunately, without reinforcement, they lack the adequate mechanical properties—in particular strength and stiffness—that would assure the safety required for such applications. Fiber reinforced composites with a thermoplastic matrix have been widely studied; however apart from [3], where resistive heating through carbon fibers was found to induce healing of E/MAA, there are no studies on fiber reinforced composites with an E/MAA matrix. Particulate E/MAA added as a healing agent to a much stiffer epoxy resin has been investigated [4,5,6], and it was found that mixing the thermoplastic particles into the thermoset matrix yields several problems. The particles are too large to fit into the narrow gaps between carbon fibers within the plies, while reducing the size to fit the plies (<10–50 μm) is not feasible because such small particles have low healing performance [4]. Stitching the composite with E/MAA filaments improves performance, particularly delamination resistance of the composite. However, the higher the stitching density, the weaker the in-plane mechanical properties will be, due mostly to the lower fiber content and damages in fibers caused by stitching. A low amount of E/MAA reduces healing properties [5]. It was shown in [7] that E/MAA reinforced with multiwall carbon nanotubes (MWCNT) can be 3D-printed. The self-healing was not affected by adding 0.1 mass% of nanoparticles, and the mechanical properties of the composite were improved. Still, the increase in modulus from 300 to 350 MPa is insufficient for widespread industrial applicability. The market of thermoplastic-based composites has been growing, mainly because they are already polymerized, and the production cycle is shorter than for thermosets. Thermoplastic materials are generally recyclable and have a lower environmental impact [8]. Immiscible blends of epoxy resin with E/MAA possess better mechanical properties than E/MAA alone, though the dominant matrix material is a thermoset. New developments and enhancements in the field of long fiber reinforced thermoplastics have identified a host of exciting applications, especially in transport and the electro/electronics sector [9]. Novel 3D-printing methods for composites with continuous carbon fibers are being developed, with a growing selection of available matrix materials. Tian et al. developed a novel method of fused deposition modeling 3D-printing and investigated the influence of processing parameters on the interfaces and performance of printed poly-lactic acid (PLA) based composites [10]. The same matrix material was used in [11], where the novel method based on fused deposition modeling allowed for direct composite production without the need for molds. Although this method is promising, the matrix material is limited to PLA. A fused filament fabrication method was applied to prepare a composite with a Nylon matrix [12]. The fused deposition modeling of an acrylonitrile-butadiene-styrene (ABS) matrix with continuous carbon fibers was investigated by Yang et al. [13]. Although 3D-printing methods are becoming more successful, the number of matrix materials and fibers content remains limited and the bending of the brittle continuous carbon fibers on product edges results in fiber damage. These methods are appropriate for small production output, high product complexity, product customization, and decentralized production [14], but not for mass production. The effect of continuous fiber and fabric orientation on hot pressed composites made of polyamide 6 (PA6) were examined by [15], where a relatively high fiber content and a successful improvement in mechanical properties were achieved, indicating that composites with sufficient fiber content resulting from pressing techniques have superior properties compared to those that are 3D-printed. Traditionally, damaged polymers were repaired through welding or patching, which was limited only to visible damage [16]. Thermoplastics with self-healing properties are of great interest as a matrix material for products where crack repairing is limited during usage or when damage arising during maintenance is poorly visible, e.g., when a tool drops onto a surface. Traditionally, damaged polymers were repaired through welding or patching which was only limited to visible damages [16]. Thermoplastics with self-healing properties are of great interest as matrix material for products where repairing of cracks is limited during usage or where barely visible damage appears during maintenance e.g., when a tool drops on a surface.

Certain production problems are still slowing the growth of the market of thermoplastic composites with continuous fibers. One issue is that thermoplastic polymers are solid at room temperature and the matrix material must be melted for composite production. Even above their melting temperature *T_m_*, thermoplastic materials are rather viscous, hindering fiber impregnation [17], while heating far above *T_m_* to reduce viscosity can affect the chemical and physical characteristics of the polymer matrix. Production parameters such as temperature, pressure, and cooling rate may influence the order of macromolecules and their oxidation and degradation, which is reflected in the material properties. Even the details in mold construction, in particular the cooling system, influence the structure and thus the final product properties.

E/MAA is a two-phase ionomer of ordered ionic clusters dispersed within a continuous semi-crystalline polymer matrix. When the temperature increases, a reduction of order and strength of the ionic clusters takes place near the order-disorder transition temperature *T_i_*. With the further increase in temperature, the semi-crystalline polymer matrix melts, even though the disordered clusters persist and continue to provide increased melt strength [18].

The healing of the E/MAA is thermally activated. Ballistic tests on this ionomer have shown that the material heals even when penetrated by a 9 mm bullet. The heat generated during the test is sufficient for the holes to heal. The healing process is divided into three stages: (i) an initial elastic response during ballistic impact, (ii) a rapid transformation into a highly elastomeric viscous polymer, and (iii) interdiffusion of polymer chains and microstructure reformation [2]. Furthermore E/MAA is sensitive to thermal history [2]. DSC measurements have proven that there is a peak about 49 °C attributed to the ordering-disordering of ionic domains present in the ionomer structure. According to [2], if E/MAA is heated above the melting temperature, cooled, and immediately reheated, the order–disorder peak is not present. As the material orders during aging at room temperature up to several weeks, the peak appears, grows, and shifts to higher temperatures. The material is physically cross-linked in two ways: (i) dominant ionic bonding and (ii) the weaker effect of acid groups that associate by hydrogen bonding.

In this study, DMA measurements on E/MAA confirmed that the behavior of the material is sensitive to its thermal history, though the effect observed here was different than that described in [2]. The peak attributed to the order-disorder of ionic domains shifted towards lower temperatures and the position, width, and intensity was dependent on the maximum temperature in the first run. We compared the effect of heating slightly below the melting temperature in the first run with the temperature at which the material begins to melt.

In this study thermoplastic composites are prepared using several heating cycles. First, polymer pellets were pressed above *T_m_* to form sheets. Then, fibers were placed between solid sheets, heated once again above *T_m_*, and repressed. Both the sheets and composites were cooled slowly in the mold at ambient temperature. To ensure uniform thermal histories, composites of E/MAA and carbon fibers were prepared and their properties compared to those of neat polymer sheets that were twice heated and pressed in the same manner as the composites. The dependence of composite properties on stress directions with respect to fibers orientation and on different fiber mass fractions was tested. Additionally, the effect of fiber position with respect to the stress neutral plane was analyzed with flexural bending tests. The high viscosity of the melt caused by persisting ionic clusters hindered the impregnation of fibers necessary for load transfer from the matrix to the reinforcement. Hence, the fibers content was rather low, though mechanical properties of the matrix were still greatly improved. The conductivity of carbon fibers enables heating the composite and thus self-healing by connecting the fibers to a direct current source as described in [3]; this feature presents high potential for maintenance.

In this study, the effect of high-velocity bullet perforation on the tensile properties of the neat polymer was tested with two calibers: 9 and 5.56 mm.

The aim of the study was to support the development of numerical constitutive models for self-healing composites. Therefore, fibers are unidirectionally oriented and the test parameters of composite specimens were selected to facilitate the validation of the numerical models.

## 2. Materials and Methods

### 2.1. Matrix Material

The ionomer Surlyn 8940 donated by DuPont was used for the preparation of transparent polymer sheets. This is a random copolymer containing 5.4 mol% of the methacrylic group. Acid groups are 30% neutralized with sodium atoms. The amount of methacrylic acid groups and neutralization level were optimized for good transparency and stiffness compared to other Surlyn types.

### 2.2. Fibers

The carbon fibers used here were commercially available HS 15/100 DL produced by G.Angeloni S. R. L., Quarto d’Altino (Italy), which are double layered (DL) unidirectional high strength (HS) fibers. The laminate of 0.095 mm thickness consists of two identical layers of Grafil 15K 1000 dtex fibers bound with glue. The amount of the carbon fibers is 94 mass% with 6 mass% is the glue and the areal mass is 100 g/m^2^. No surface treatment was performed after purchasing.

### 2.3. Processing Parameters

In the present study, neat E/MAA sheets were reinforced with UD carbon fibers by hot compression molding in a vacuum bag. Prior to the preparation of the sheets, pellets were dried at 50 °C for at least 24 h. The melting interval was determined using a polarizing microscope with a heating system in the procedure described in EN ISO 3146:1985. Melting started at 91 °C and ended at 93 °C, which is in agreement with the melting interval declared in the data sheet given by DuPont. Given that there are persisting disordered ionic clusters in the melt of E/MAA, the processing temperature needed to be markedly higher than the melting interval.

Prior to selecting processing parameters, a short investigation of the different parameters was conducted. The problems of trapped bubbles in the polymer matrix, sliding fibers, and impregnation were reduced by varying the temperature, compression pressure and duration of pressure application (Table 1). The first composite (Case 1) was prepared at 130 °C with the goal of minimizing the heat effect on the polymer. The resulting laminate had many trapped bubbles. Increasing the temperature led to fewer bubbles; however, the fabric slid when pressure was applied (Case 2). Extending the annealing time and reducing both the intensity and duration of the pressure applied gave the best results (Case 3), as the bubbles had enough time to leave the matrix and the melt impregnated the fibers. One plate was prepared with only manual pressing instead of using the machine press. These parameters were comparable to those described in [3]. In this way, the fewest bubbles were visible, though the bonding of carbon fibers with the matrix was very weak (Case 4).

The composites prepared with the parameters described in the Case 3 were selected for testing in the present study.

### 2.4. Preparation of Composites

UD fibers were placed between previously pressed solid polymer E/MAA sheets in a steel mold. The mold was placed in a vacuum bag and the air was evacuated for 30 min at ambient temperature using a rotary vane pump. The mold in the vacuum bag was placed in the press, where the polymer was melted at 160 °C. Gases were evacuated from the melt for 20 more minutes to avoid gas bubbles. Following this, a pressure of 10 bar was applied for 2 min. The pressure in the vacuum bag was 0.1 bar throughout the process. Finally, the mold was slowly cooled down at ambient temperature. Composites with single or double layers of fibers are prepared, where the fiber content was 2 mass% and 2.5 mass%, respectively. Fiber content was measured using the resin burn-off test. Composite specimens were heated to 550 °C for two hours in air to decompose the matrix material. According to [19], these conditions caused very little oxidation and mass loss to carbon fibers.

For comparison with the composites, the non-reinforced matrix material was prepared in the same manner as the composites, i.e., two sheets were pressed separately and then pressed together under the same conditions, to ensure uniform thermal histories between the matrix material and the composite. The thickness of sheets was regulated using a steel frame as a distance holder in the mold. The thickness of polymer sheet was 1.6 ± 0.05 mm and for the composite it was 2.3 ± 0.15 mm.

### 2.5. Dynamic Mechanical Analysis (DMA)

The goal of DMA was to determine the impact of thermal history on the mechanical properties of E/MAA. For these tests, the polymer sheets were not heated twice during the preparation as described above. Stripes (approximately 20 mm × 3 mm) were cut from the approximately 1.6 mm thick polymer sheet, with a jaw span of 8.3 mm. Two successive runs were performed on polymer samples, starting at 25 °C. The upper temperature limit in the first cycle varied: in the first case it was 89 °C, which is near the melting start temperature of 91 °C, and in the second case it was 98 °C, which is above the melting end temperature *T_m_*. Even at 5 °C above *T_m_* there is still enough elastic response for the DMA to be performed, thanks to the ionic clusters in the melt. The tests were performed on the TTDMA, Triton Technology in tensile mode, with a frequency of 1 Hz, amplitude of 0.002 mm, and a heating rate of 2 °C/min.

### 2.6. Flexural Properties and Apparent Interlaminar Shear Strength

Flexural and tensile properties were tested on a universal testing machine with a crosshead speed of 7 mm/min. The tests were performed on samples with single or double layers of reinforcement and compared to those of the neat polymer. The double layer composite was tested in three directions (0°, 45°, and 90°) with respect to the UD fiber orientation. Flexural properties were obtained from the three-point bending test in accordance with EN ISO 178:2003. The apparent shear interlaminar strength was tested according to EN ISO 14130:1997. The setup of the shear strength test is the same as for the three-point bending test, with a difference in span, i.e., 16 times the average height of the tested samples in the bending test vs. 5 times in the apparent interlaminar shear strength test.

### 2.7. Tensile Properties

All tensile properties were tested according to EN ISO 527-4:1997 with dog-bone samples and the crosshead speed was 7 mm/min. The only exception was the test on the E/MAA before and after ballistic impact. The bars with one damaged spot included in the middle were cut as simple bars around scars that remained in the middle, with a width of 15 mm, thickness of 4 mm, and length of 130 mm. The initial controlled length *l_0_* was 50 mm. The tests were performed with the crosshead speed of 50 mm/min.

### 2.8. Ballistic Tests

Ballistic tests were performed on neat E/MAA. To avoid target deformation, 4 mm thick plates were prepared. Tests were performed with two calibers (9 and 5.56 mm) outdoors under winter conditions, at an air temperature of about 0 °C.

Tensile tests were conducted on E/MAA before and after ballistic impact with the 9 mm caliber.

## 3. Results

### 3.1. Dynamic Mechanical Analysis

The DMA is a powerful tool for observing structural changes in a material and their influence on mechanical response. The effect of thermal history can be observed through changes in the loss factor tan δ and storage modulus *E*’ in temperature scans.

**Figure 1:**
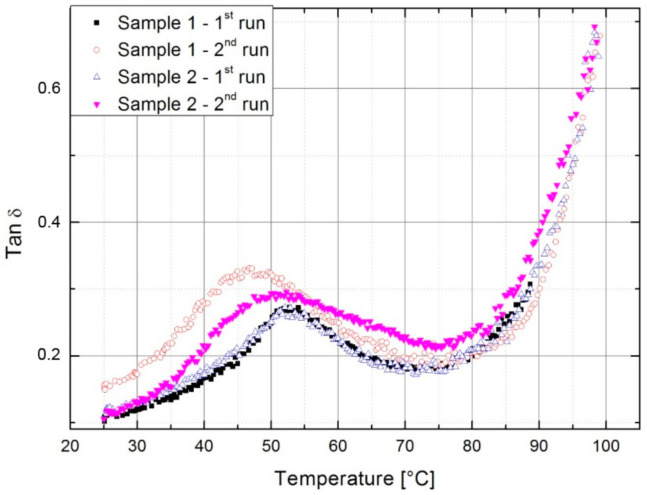
Tan δ of the neat polymer. In the first run the samples were heated either to 89 °C (Sample 1) or 98 °C (Sample 2).

The dependence of tan δ on temperature is presented in Figure 1. The first peak in the spectrum measured from the ambient temperature is attributed to the order-disorder transition of ionic domains present in ionomer structure [2], who reported in [2] that the peak disappeared when heated above the melting point, cooled, and then reheated. However, the DMA tests performed here did not give this result. Regardless of whether the maximum temperature in the first cycle was below or above the melting interval, the peak measured in the second cycle broadened and shifted to lower temperatures. The peak position was dependent on the maximum achieved in the first run. After cooling from 98 °C, it was positioned nearer to thee values in the first cycle. This means that the first run to 89 °C which is less than *T_m_* had a greater effect on the order-disorder peak. The material is physically less cross-linked with greater mobility of molecules participating in the relaxation process.

**Figure 2:**
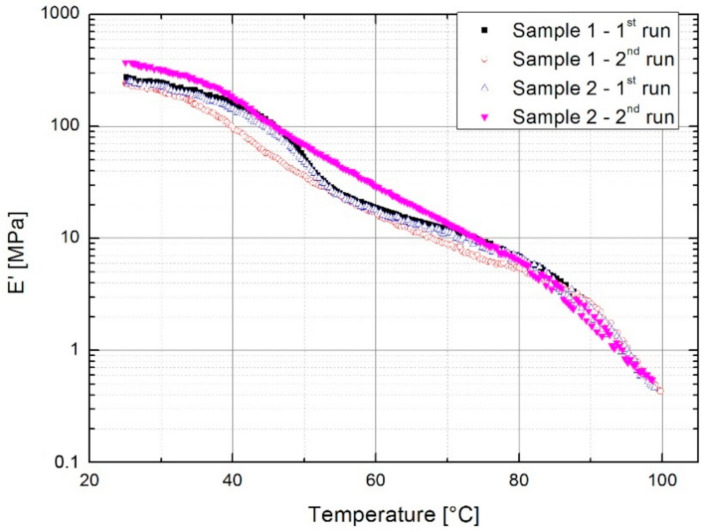
Storage modulus of the neat polymer. In the first run, samples were heated either to 89 °C (Sample 1) or 98 °C (Sample 2).

The reduction of cross-linking density after heating below *T_m_* (Sample 1) is also evident in Figure 2, where the modulus in the second run was lower, and the shape of the curve more continuous than in the first cycle. Near *T_m_*, the moduli and tan δ are of comparable values since the lower viscosity allows for the rearrangement of molecules (Table 2). The measurement at which the *T_m_* is reached (Sample 2) had a much weaker effect since the higher temperature and lower viscosity enabled reordering of molecules. Compared to the first run the resulting storage modulus in the second run was higher below 75 °C and the continuous shape remained observable. The increase in modulus after cooling from *T* > *T_m_* was due to the development of secondary polyethylene crystallites during the first run, where the material had already melted and cooled from the melt [20,21]. Just below the melting of the primary crystallites, the ionomers are described as two-phase composites of crystallites and ionically cross-linked rubber [20], hence the secondary crystallites melt below *T_m_* and the modulus values approach the same values as in the first cycle (Table 2). The effect of melting of smaller secondary crystallites is also observed in tan δ, since melting starts at lower temperatures. The existing secondary crystallites melted in the first run of both samples; however, they did not have time to form in the case where the *T_m_* was not reached in the first run.

Table 2 provides an overview of the storage moduli at certain temperatures taken from Figure 2. Heating Sample 2 to the melting temperature and slow cooling increased the modulus at 25 °C by 34.5%, which was observed in the second run. Above 75 °C, the values for all measurements are comparable.

The mechanical spectra show that the thermal history had a significant influence on the matrix material. In the production of composites, the matrix material was heated twice, the first time for polymer sheets preparation and the second time for composite production. Therefore, in the comparison of the composite with the matrix, the matrix material was also heated twice in the same manner as the composite.

### 3.2. Flexural and Tensile Properties

When the samples are bent, the stress is compressive at the top and it is tensile on the bottom. Near the midplane, there is a neutral plane where there is neither the compressive nor tensile stress. In the case of the single fibers layer, the reinforcement is placed between two polymer sheets near the neutral plane, while in the case of the double layer, the fibers are positioned symmetrically with the respect to the neutral plane. Hence, one ply is in the tension region and the other in the compression region, with approximately the same offset from the neutral plane. Due to the low modulus and thus large deflection, the neat polymer samples did not break during the bending test, and therefore flexural strength could not be tested. The same behavior was observed for the composite with the single layer reinforcement, since the reinforcing fibers were near the neutral load plane.

**Figure 3:**
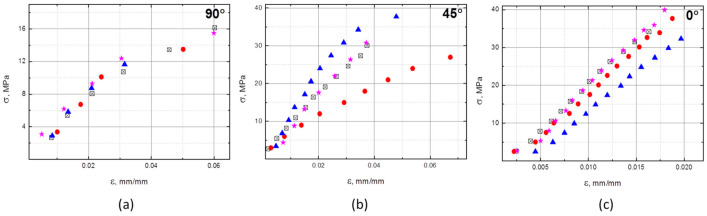
Results of flexural bending tests on composite reinforced with a double layer of fibers in three directions: (**a**) in the 90°, (**b**) in the 45° direction and (**c**) in the 0°direction, with respect to UD fibers orientation (four samples tested for each direction, each data set is presented with a different symbol).

**Figure 4:**
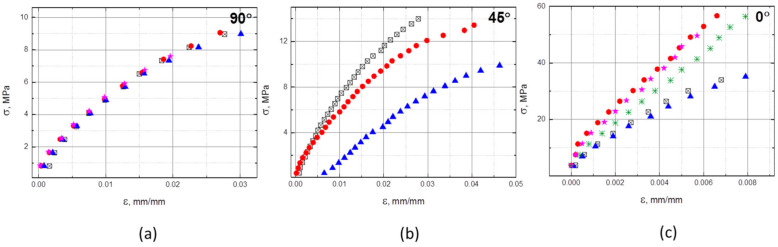
Results of tensile tests on the double layer reinforced composite in three directions: (**a**) in the 90°, (**b**) in the 45° direction and (**c**) in the 0°direction, with respect to UD fibers orientation (a data set for each tested specimen is presented with a different symbol).

**Figure 5:**
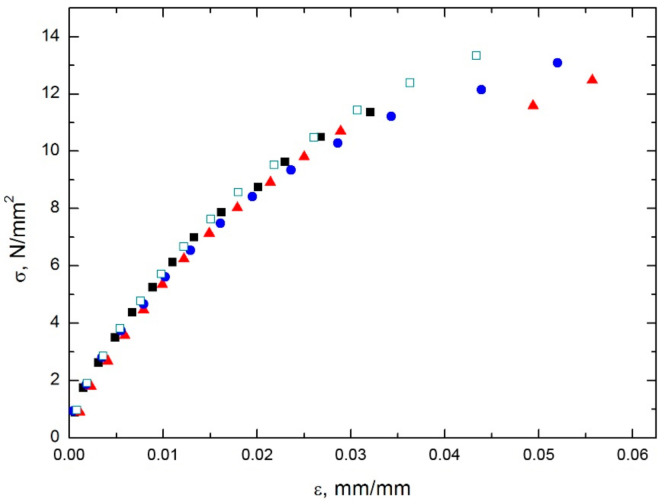
Results of tensile tests on the neat polymer.

The stress-strain measurements for the composites reinforced with a double fiber layer in all three directions are shown in Figure 3. Deflection data could not be measured completely, due to limitations of the testing machine. Therefore, the maximum stress visible in diagrams is not equal to the flexural strength shown in Table 3. The strength values for the neat resin and the single layer composite were not determined in the experiment and are therefore not included in Table 3. The same limitation exists in the data presented in Figure 4 and Figure 5.

The flexural modulus of E/MAA is 196.9 MPa and a single layer of UD fibers in 0° direction improved this by only 22% (Table 3). However, stiffness was more effectively increased with the double layer of fibers pressed between three matrix sheets, although both composites had a low mass fraction of fibers. This effect is caused by the placement of fibers further from the neutral midplane and thus in a region of higher stresses, thus playing a more important role. The flexural modulus in the 0° direction was 11.7 times higher than that of the matrix material, at 45° it was 3.9 times higher, and at 90° it was 2.2 times higher. The moduli of the composite with a 45° orientation were rather scattered and the testing results for this direction were the least reliable.

The flexural strength for 0° was the highest of all three directions, though a higher impact of fibers orientation was expected. The difference between the 45°and 0° orientation was 18%, which could be due to the stress (*σ*) as calculated according to the Euler–Bernoulli Equation as:

*σ* = 3*Fl*/(2*bh*^2^), (1)

where *F* is the force, *l* is the span, *b* is the width, and *h* is the height. The 45° direction is anisotropic and, in addition to tensile and compression stress in the sample, includes a shear stress, and hence this case is not well described by the equation.

The tensile tests presented in Figure 4 and Figure 5 and Table 4 prove that even the low fiber content contributed very efficiently to the mechanical properties in the tensile direction when the load was applied in the fibers’ direction (0°). The modulus was 13 times higher than that of the neat polymer and the strength was 4.2 times higher. However, the tensile properties in the transverse direction (90°) degraded in comparison with those of the matrix material, especially tensile strength which decreased by 36.8% while the tensile modulus decreased 5%. The fibers barely take over the load and the effect of flaws in the composite is dominant. When fibers were rotated by 45°, the same effect of scattered data was observed as in the flexural tests. The average tensile modulus increased by 21% and the tensile strength by 28.9%.

The apparent shear interlaminar strength was tested only for the 0° orientation. The values were calculated as:

*τ* = 3*F*/(4*bh*). (2)

The apparent shear interlaminar strength was calculated as 5.4 MPa for the single layer composite and 4.7 MPa for the double layer composite. These similar values indicated that the prepared composites are of comparable quality.

### 3.3. Ballistic Tests

**Figure 6:**
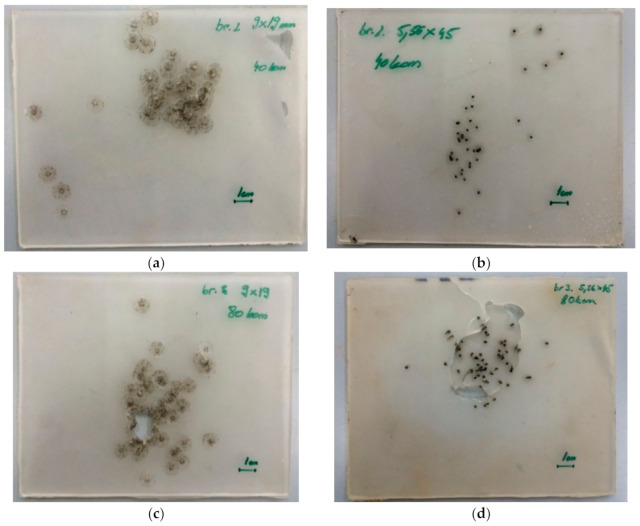
Ballistic tests with different calibers, (**a**,**c**) 9 mm and (**b**,**d**) 5.56 mm. The tests (**a**,**b**) were performed with 40 shots and (**c**,**d**) with 80 shots (the scale in the lower right corner shows 1 cm.

**Figure 7:**
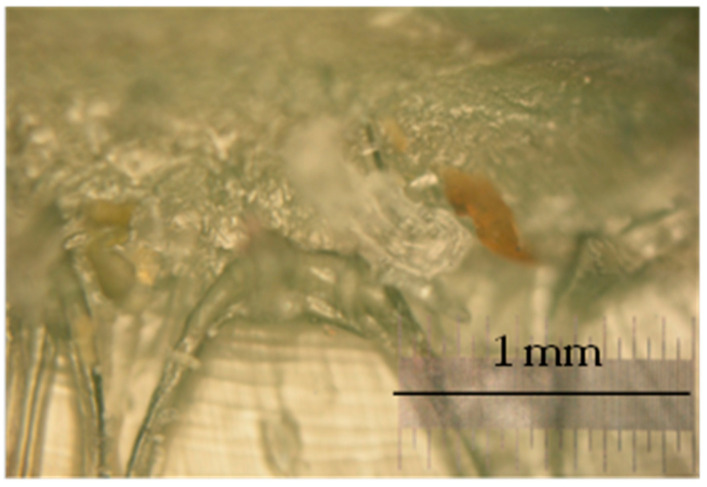
Scar after the ballistic test—transparency of the material is lost and there are visible copper particles.

Outdoor ballistic tests at an air temperature of 0 °C showed that the material heals instantaneously. After the ballistic tests, scars were visible, and some impurities remained in the targets but were not analyzed (Figure 6). In the case of 9 mm bullets with copper jackets, copper particles were visible under a light microscope (Figure 7). The material managed to self-heal after 40 shots, yet with 80 shots, target disintegration occurred.

**Figure 8:**
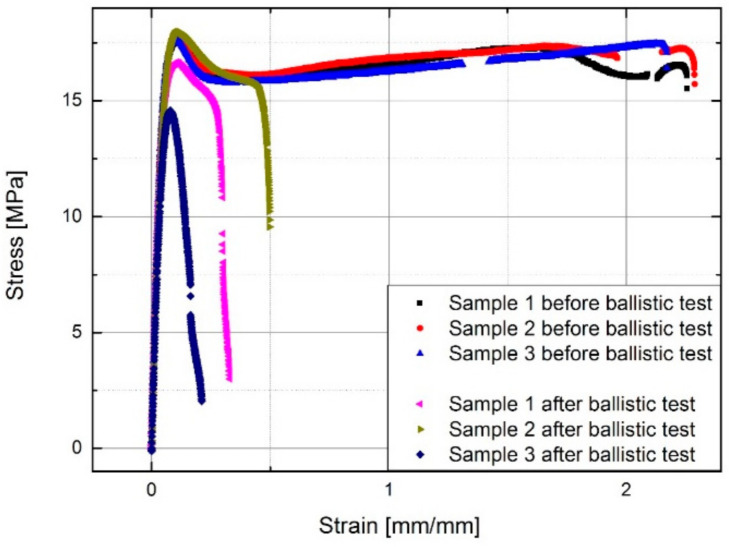
Tensile tests on samples before and 9 mm caliber ballistic tests.

The tensile properties of E/MAA were tested before and after the 9 mm caliber ballistic test. The width of testing bars was 15 mm, or about 60% of the cross-section surface was damaged. The samples broke at the site of the scar. Figure 8 shows that the tensile modulus was not affected. However, the initial controlled length was 50 mm and the scar affected only 18% of the total controlled length. The tensile strength was reduced, and the values were scattered, though the fracture strain was mainly affected by the damage. The weak reproducibility of results was partially due to sample imperfections and the impurities included in the holes, and therefore, it is difficult to predict the strength and elongation at the break after healing.

## 4. Discussion

The experimental investigation of appropriate processing parameters was based on previous studies, where neat E/MAA was processed either at 150 °C [2], or at 160 °C [22] and composites with E/MAA matrix reinforced with carbon fibers were prepared at 160 °C [3]. In [7], E/MAA reinforced MWCNTs was extruded at 135 °C.

The DMA tests showed that thermal history affected the structure and properties of E/MAA. Heating to a sufficiently high temperature, above *T_m_*, and allowing time for molecules to reorganize resulted in the secondary crystallization of polyethylene as described in [20,21]. This was reflected in the changes in the temperature scans of damping properties and storage modulus. In this study, the disappearance of the relaxation peak could be attributed to the lack of an order-disorder transition of ionic domains as described in [2]. The position and intensity were found to be dependent on the maximum temperature achieved in the previous heating.

Disordered ionic clusters in the E/MAA melt contributed to the elastic response to mechanical stress. The resulting viscosity created an obstacle for the impregnation of continuous fibers in E/MAA based composites, making it a challenge to prepare a composite with a reasonable fiber content. Nevertheless, even 2.5 mass% of carbon fibers substantially improved the mechanical properties of the matrix, particularly for stresses in the 0° direction. Tensile modulus and strength in the 0° direction increased 13 times and 4.2 times, respectively. Zhang et al. reinforced PA6 with 30 vol% or approximately 40 mass% carbon fibers using a similar method and reported an increase in the modulus by approximately 50 times and in tensile strength by 13 times [15]. Their reinforcement fraction was 16 times higher than in the present study, though their mechanical properties were not improved 16 times. Mixing fibers with E/MAA particles milled to a size of 10 to 50 µm prior to lamination could improve impregnation, thus enabling a higher fiber fraction that would be much more efficient. Melting the matrix material would also melt the particles and since the matrix and particles are of the same material, there should be no issues in binding and obtaining a continuous matrix. This would thereby sidestep the issue of reduced healing efficiency of small particles described for epoxy-based composites [4] with E/MAA mixed as a healing agent. When tensile stress was applied to samples with reinforcement rotated by 90° with respect to load, the mechanical properties degraded, indicating the presence of voids between fibers and the matrix. Higher impregnation should improve the properties even in the worst case when the load is perpendicular to fiber orientation. A detailed analysis of these voids is needed.

Novel 3D-printing methods are excellent tools for complex, customized, and small series products. However, they still result in composites with weaker mechanical properties. The direct 3D fabrication of PLA reinforced with 6.6 mass% of continuous carbon fibers improved the tensile modulus of PLA by 599% and strength by 435% [11].

A limited mass fraction of fibers in the composite prepared by pressing leads to a heterogeneous cross-section of the material. A superior composite is achieved when fibers are placed near the composite surface, instead of in the middle near the neutral stress plane. Comparing the results for flexural behavior of the composite with single or double layers (Table 3), it is obvious that when the cross-section is non-uniform, the mechanical properties are more dependent on fiber position than on fiber content. Since the fibers in the cross-section of specimens were not uniformly distributed, these flexural properties should be understood as reference values and cannot be compared to other studies. Nevertheless, in a study where PLA was reinforced with continuous carbon fibers using the fused deposition modeling 3D printing process (FDM), a composite with the lowest fiber fraction of approximately 7.5 mass% showed a flexural modulus of 7.5 GPa and flexural strength of 130 MPa [10]. In the present study, with a 2.5 mass%, the flexural modulus was 2.3 GPa and the strength was 39.9 MPa. The properties of the composite based on ABS with a 10 mass% of continuous carbon fibers produced by FDM compared to those of injection molded material showed that the interface performance of the former were inferior [13].

E/MAA can be 3D-printed, however, there are no published studies on 3D-printed E/MAA with continuous fibers, only reports concerning 0.1 mass% MWCNTs where the tensile modulus increased by only 16% [7].

Ballistic tests cause local damage with heat development in the material that is necessary for self-healing. The molecular structure was affected by heating, cooling, mechanical impact, and by the impurities present within the material. Tensile tests after ballistic impact showed that the fracture occurred at the site of bullet penetration through the material, showing a reduction in the mechanical properties of the self-healed material. The results indicate that the ballistic tests did not reduce elastic modulus. Yet, yield point, tensile strength, and particularly elongation at break were reduced and the test results were scattered. The outcome in properties depends on the thermal history of E/MAA and impurities of the materials, and therefore, it is difficult to predict the properties of the healed material in application. Since only a part of the tested samples self-healed, the conducted tensile tests were not representative. They only indicate that the site of bullet penetration was the weakest in tensile tests. Future mechanical tests on E/MAA and continuous carbon fiber reinforced E/MAA are required, with a larger fraction of self-healed matrix material and with an impact that does not leave impurities in the material, under controlled heating conditions using resistive heating through carbon fibers, as described in [3].

Flexural tests on self-healed E/MAA were not performed in this study, since the samples did not break during the three-point bending test prior to the ballistic testing. Additionally, during the three-point bending test, the force was applied exactly in the middle, at the site of the scar. The flexural modulus was highly influenced by the behavior of the intact E/MAA. This suggests the need for development of a new method of performing this study using a higher fraction of healed material and without material impurities.

## Figures and Tables

**Table 1:** Parameters for preparation of the composite plates with a single fiber layer. Testing was performed on composites made using the parameters applied in Case 3.

Parameter	Case1	Case2	Case3	Case4
Annealing temperature [°C]	130	160	160	160
Annealing time [min]	15	15	20	15
Pressure [bar]	40	40	10	Manual pressing
Compression time [min]	15	15	2	-

**Table 2:** Storage modulus of the neat polymer at different temperatures. All values in MPa.

Modulus	*E*’ (25 °C)	*E*’ (40 °C)	*E*’ (55 °C)	*E*’ (75 °C)	*E*’ (80 °C)	*E*’ (89 °C)
Sample 1 1st run to 89 °C	275.2	162.5	25.4	9.2	6.9	3.0
Sample 1 2nd run	241.8	94.7	24.0	6.4	5.5	2.9
Sample 2 1st run to 98 °C	250.3	142.9	24.2	8.1	6.7	2.8
Sample 2 2nd run	370.2	182.0	43.6	8.8	6.3	2.5

**Table 3:** Flexural modulus *E_f_* and flexural strength *R_mf_* of the four samples of the double layer (2L) composite cut in three directions with respect to the fibers orientation and the modulus for single layer (1L) composite and for neat polymer samples. All values are in MPa.

	2L—90°		2L—45°		2L—0°		1L—0°		Neat Polymer	
	*E_f_*	*R_mf_*	*E_f_*	*R_mf_*	*E_f_*	*R_mf_*	*E_f_*	*R_mf_*	*E_f_*	*R_mf_*
1	407.6	17.5	751.3	34.2	2375.8	38.1	236.9	-	206.9	-
2	479.6	13.5	382.6	28.5	2274.5	40.2	232.2	-	119.2	-
3	459.5	13.1	1046	39.4	2031.8	33.5	250.3	-	264.5	-
4	362.4	15.5	871.0	33.0	2560.8	47.7		-	196.8	-
M	427.3	14.9	762.7	33.8	2310.7	39.9	239.8	-	196.9	-
SD	52.9	2.0	280.8	4.48	220.5	5.92	9.4	-	73.2	-

**Table 4:** Tensile modulus *E_f_* and tensile strength *R_mf_* of the four samples of double layer (2L) composites cut in three directions with respect to fibers orientation, and of the neat polymer. All values are in MPa.

	2L—90°		2L—45°		2L—0°		Neat Polymer	
	*E_t_*	*R_mt_*	*E_t_*	*R_mt_*	*E_t_*	*R_mt_*	*E_t_*	*R_mt_*
1	463.0	9.5	767.1	22.6	4835.9	34.7	515.4	14.9
2	445.6	10.8	604.1	21	7047.7	78.6	495.5	15.3
3	486.9	9.6	413.3	15.1	7241.7	68.7	467.5	15.3
4	468.9	8.3			6363.8	72.2	487.6	15.2
M	466.1	9.6	594.8	19.6	6372.3	63.6	491.5	15.2
SD	17.0	1.0	177.1	4.0	1091.3	19.7	19.8	0.18

## Data Availability

Data is contained within this article.
